# It's All in the Timing: Too Much E2F Is a Bad Thing

**DOI:** 10.1371/journal.pgen.1002909

**Published:** 2012-08-16

**Authors:** Brandon N. Nicolay, Nicholas J. Dyson

**Affiliations:** Massachusetts General Hospital Cancer Research Center, Charlestown, Massachusetts, United States of America; University of Washington, United States of America

E2F is one of the best-known cell cycle–dependent transcription factors. The family of E2F proteins have been highly conserved during evolution and function as both transcriptional activators and repressors. In most animals, E2F-mediated activation is a key driver of cell proliferation, and deregulation of E2F is found in most types of cancers. In addition to its proliferation-promoting properties, elevated E2F also makes cells sensitive to apoptosis and tumor cells acquire additional mutations, such as mutations in the p53 pathway, to suppress this deleterious activity. Part of the explanation for this aspect of E2F biology is that E2F proteins bind directly to the promoters of several pro-apoptotic genes, and E2F-mediated activation is thought to help to enhance levels of apoptosis, particularly in response to DNA-damaging agents [1], [2].

The best-characterized mechanism of E2F regulation is the reversible ability of the retinoblastoma tumor suppressor protein (pRB) to bind to activator E2Fs and inhibit their activity. However, organisms have evolved additional mechanisms to limit E2F activity. In *Drosophila*, the level of the lone activator E2F protein (dE2F1) oscillates dramatically in a developmentally regulated manner [3], [4]. In previous studies, Duronio and colleagues discovered that this regulation is due to a precisely controlled pattern of dE2F1 degradation that is mediated via a PCNA-interacting protein motif in dE2F1, termed a “PIP-degron” [5]. The PIP-degron promotes the proteolysis of dE2F1 in S-phase cells through an interaction with DNA-loaded PCNA, coupled with the recruitment of the CRL4^Cdt2^ E3 ubiquitin ligase. The importance of this regulation was revealed by experiments showing that expression of a mutant dE2F1 protein that lacked this motif, and that is stable into S-phase (dE2F1^Stable^), leads to expedited cell cycle progression, apoptosis, and developmental defects.

**Figure 1:**
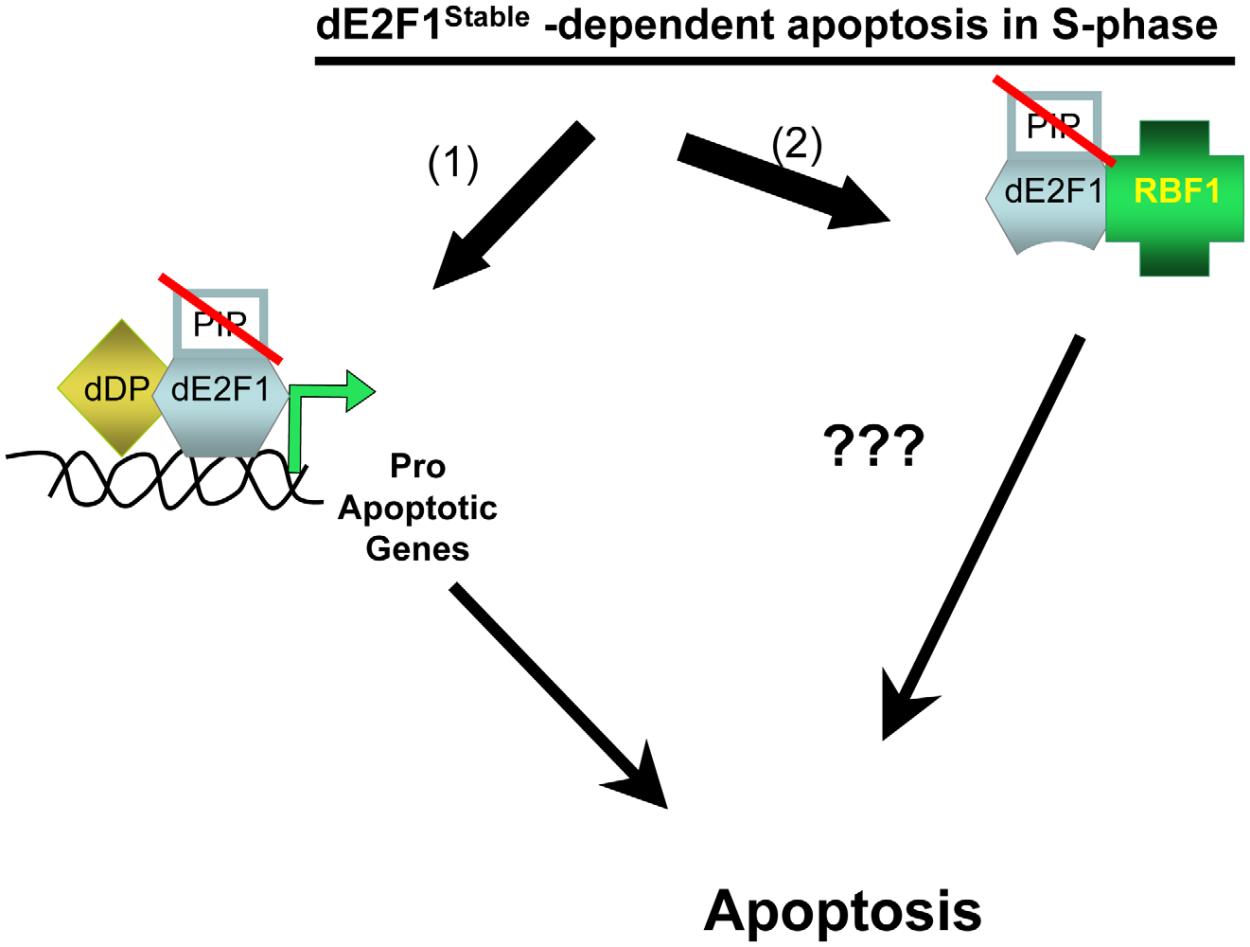
Stabilized dE2F1 can induce apoptosis through transcriptional and non-transcriptional mechanisms. (1) During S-phase, a dE2F1 protein without a PIP-degron but with an intact DNA–binding domain will promote apoptosis through transcriptional activation of pro-apoptotic genes, such as *hid*. (2) A dE2F1 protein without a PIP-degron and lacking an intact DNA-binding domain can promote apoptosis through an unknown mechanism that requires a physical interaction with the RBF1 protein; this unknown mechanism is also dependent upon *hid*.

In the present study [6], Davidson and Duronio have carried out a meticulous analysis of a panel of dE2F1 transgenic lines and have identified the functional domains that enable dE2F1 to promote apoptosis when it persists in S-phase cells. That stabilized dE2F1 causes apoptosis was not unexpected, but the authors report the surprising finding that this property of dE2F1 does not require its DNA-binding activity but instead requires C-terminal sequences that contain the RBF1-binding domain. A key result in this set of experiments is the observation that expression of a mutant dE2F1 protein with lesions in both the PIP-degron and the DNA-binding domain (dE2F1^DBD/Stable^) induces apoptosis when expressed in eye or wing imaginal discs, even though this protein lacks the ability to activate E2F-dependent gene expression or to drive cell proliferation. Collectively, the results from the panel of mutants suggest that the presence of dE2F1 in S-phase can promote apoptosis via a mechanism that is distinct from dE2F1's traditional role as a transcriptional activator (Figure 1).

These observations raise the question of how dE2F1^DBD/Stable^ triggers apoptosis in S-phase cells. One potential explanation might be that dE2F1^DBD/Stable^ acts by sequestering RBF1, a *Drosophila* homolog of pRB. Indeed, it seems likely that some of the properties of stabilized dE2F1 are mediated via RBF1 since a mutant form of dE2F1 with an impaired ability to bind to RBF1 (dE2F1^Stable/RBmut^) gives reduced levels of apoptosis, and a truncation mutant that completely eliminates the RBF1–binding domain (dE2F1^Stable/Trunc^) is unable to induce apoptosis. Moreover, apoptosis induced by both dE2F1^Stable^ and dE2F1^DBD/Stable^ is dependent on the gene dosage of *hid*, a previously characterized dE2F1 target gene that is also important for apoptosis resulting from the inactivation of *rbf1* [7], [8]. However, Davidson and Duronio show that another deletion mutant of dE2F1, which lacks the DNA-binding domain but maintains binding affinity for RBF1 (dE2F1^306–805/Stable^), fails to induce apoptosis. This indicates that the ability of stabilized dE2F1 fragments to bind to RBF1 is not, by itself, sufficient for apoptosis. These results suggest that there is an as yet to be characterized property of dE2F1 that promotes apoptosis in S-phase cells, that this mechanism does not require the DNA-binding domain of dE2F1, and although it likely involves dE2F1/RBF1 complexes, it is not a simple sequestration of RBF1.

What is the physiological significance of these observations? High levels of dE2F1 are not normally seen in S-phase cells; Davidson and Duronio propose that the pro-apoptotic activity of stable dE2F1 reveals a homeostatic mechanism that cells use to sense the inappropriate presence of elevated dE2F1 and to eliminate cells that might be dangerous. This is a very attractive idea given the importance of deregulated E2F in cancer cells and the potential utility of a mechanism that can trigger apoptosis in cells with elevated E2F activity.

In the future, additional studies are clearly warranted to better understand this property of dE2F1. Immunostaining experiments show that dE2F1 levels fall abruptly as cells enter S-phase in imaginal discs, and it is evident that persistence of dE2F1 into S-phase is a very rare event in wild-type larvae. Moving forward, it will be important to identify a physiologic context in which this homeostatic mechanism is utilized; and it will be important to demonstrate that it can be activated by the endogenous dE2F1 protein, rather than by transgenes driving the ectopic expression of mutant fragments of dE2F1. A major question will be whether a similar mechanism is operative in mammalian cells. Although the PIP-degron is not conserved in E2F proteins in other species, other interactions have been described that lead to the turnover of mammalian E2Fs after the G1/S transition [9], [10]. It will be extremely exciting if a conserved mechanism of E2F action exists that occurs independently of E2F's ability to bind to DNA and that enables deregulated E2F to trigger cell death.
